# Vertical distribution and analysis of micro-, macroelements and heavy metals in the system soil-grapevine-wine in vineyard from North-West Romania

**DOI:** 10.1186/s13065-015-0095-2

**Published:** 2015-04-12

**Authors:** Florin-Dumitru Bora, Claudiu-Ioan Bunea, Teodor Rusu, Nastasia Pop

**Affiliations:** Department of Horticulture and Landscaping, Faculty of Horticulture, University of Agricultural Sciences and Veterinary Medicine, 3-5 Mănăştur Street, 400372 Cluj-Napoca, Romania; Department of Technical and Soil Sciences, Faculty of Agriculture, University of Agricultural Sciences and Veterinary Medicine, 3-5 Mănăştur Street, 400372 Cluj-Napoca, Romania

**Keywords:** Micro-, Macroelements, Heavy metals, Soil-grapevine-wine, FAAS technology, *Vitis vinifera* L, Grapevine cultivars

## Abstract

**Background:**

The determination of micro-, macroelements and heavy metals in the soil-grapevine-wine system is extremely important for the wine industry, the grape and wine quality, and also for consumer health. The quantitative analysis of 10 elements: Na, Ca, Mg, Fe, Cu, Zn, Pb, Cd, Ni, Co were made in soil at different depths and also in grapevines (leaves and canes). For grape juice and wine there were analyzed the concentrations of Cu, Zn, Pb, Ni and Cd on three cultivars Fetească albă, Fetească regală and Riesling italian, located in Turulung vineyard, NW Romania. All the elements were detected using flame atomic absorbtion spectrometry (FAAS).

**Results:**

Only the Cu concentration $\left(\overline{x}=479.64\kern0.5em \mathrm{mg}/\mathrm{kg}\right)$ has higher values than the maximum limit admitted (20 mg/kg). The concentrations of micro-, macroelements and heavy metals in aerial parts of grapevine cultivars occur in the following order: Ca > Na > Mg > Fe > Cu > Zn > Ni > Pb > Co > Cd in canes and leaves. Cu, Pb, Ni and Zn concentration levels decreased in wine compared to grape must, possibly forming insoluble components that can be removed through sedimentation together with yeasts and lees during fermentation. Cd was under the limit of detection. Heavy metals detected in Romanian wines were below the recommended health limits of the International organization of wine and vine (O.I.V.).

**Conclusions:**

In soil, all the elements studied were under the maximum limit admitted, except, elevated concentrations of Cu. These high values obtained could be an effect of different Cu treatments in vineyards. In canes and leaves, Cu, Zn, Pb, Cd, Ni had higher concentration levels than in grape juice (must) and wine. Conversely, the metal acumulation of wines obtained by micro-vinification process (in the laboratory) are lower than in must.

## Background

Different wine and grape cultivars have appeared over the centuries of cultivation, according to the skills and tastes of grape growers and wine makers [1]. *Vitis vinifera* grapes are frequently used for wine production around the world, especially in Europe. All over the world, approximately 80% of all grapes are used in winemaking and 13% are consumed as table grapes [2]. The quality and quantity, origin, aroma characteristics and health safety of grape and wine consumption are influenced by environmental and anthropogenic factors [3-5]. Between them, environmental factors are region, orography, eco-climate (e.g. temperature, precipitation, wind, etc.), type and composition of soil [4,5]. Of anthropogenic factors responsible for pollution of vineyards is highlighted inputs resulting from the use of fertilizers and metal-based pesticides, chemical sprays, organic manures, industrial emissions, transportation and municipal wastes [6-9] and winemaking technology and storage [10].

Industries from Romania like metallurgy, mining activity (related with the flotation and smelters), energy and fuel production, organic and inorganic pesticide and fertilizer industry (also their application) release wastes containing different heavy metals, in soil and other environmental components [11,12].

Generally vineyard soils are very degraded and more ready to contamination. In this context, heavy metal pollution of vineyard soils is a major environmental problem that can affect plant productivity, food quality and human health. Some metals like Se, Fe, Cu, Zn and Mg are essential metals since they play an important role in biological systems, while Al, Pb, Ni and Cd are non-essential metals as they are toxic even in trace amounts [13,14].

Because most pollutants (in the form of solids or gases) can migrate through wind, surface water or groundwater from source of pollution to vineyards, the number of contaminated areas grows larger every year, and heavy metal pollution is a problem in some viticultural regions from Romania [6,11] and other European countries like Croatia [4], France [9], Greece [15] and Bulgaria [16].

Naturally, around 0.9 million ha of soils, are affected by chemical pollution and 0.2 million of them by excessive pollution. Between all the soil pollution contaminants, heavy metals (Cu, Pb, Zn and Cd) or acid precipitation represent agrresive effects on soil and ussually can be found in areas like Baia Mare, Zlatna or Copşa Mică [17]. These regions from Middle and Northwest Romania are well-known contaminated areas with heavy metals [18,19]. Particularly in Baia Mare, two metallurgical factories (Romplumb and Cuprom) are the main sources of pollution for the city [12,20] and also for the neighboring agricultural areas.

The aim of this study is to obtain the overview on micro-, macroelements and heavy metals in the northwestern Romanian vineyard soil, aerial parts of vines, grape juice and wine. Our work evaluated 10 elements: Na, Ca, Mg, Fe, Cu, Zn, Pb, Cd, Ni, and Co in 3 grapevine cultivars Fetească regală, Fetească albă and Riesling Italian cultivated in the Turulung area, located at 57 km (NW) from Baia Mare. Detection of minerals and heavy metal concentrations in the system soil-grapevine-wine has been conducted using flame atomic absorption spectrophotometry (FAAS). FAAS is the most common used technique for trace metals (with relatively high concentrations) quantification in soil, vegetal samples and wine. For some elements present in low concentrations, electrothermal atomic absorption spectrometry (ETAAS) or graphite furnace atomic absorption spectrometry (GFAAS) are used [5,14].

The objectives of the study were to: (i) determine micro-, macroelements and heavy metals in soil, canes and vine leaves, grape juice (must) and wines (young wines - after fermentation); (ii) reveal mobility and bioavailability of trace metals in system soil-grapevine-wine; and (iii) compare obtained data with previous studies.

## Results and discussion

### Micro-, macroelements and heavy metals in soil

The characteristic type of soil from Turulung area is Preluvosoil (EL) from Luvisols (LUV) class. The soil is characterized by a pH from 6.0 to 6.12, a content of 1.3-3.5% humus, 20–35 ppm mobile P, 130–220 ppm mobile K, 1.0-2.5 N index, and redox potential between 361–397 mV. Texture is clay-loam, granular structure, well-formed, and after 110–140 cm depth of horizon, structure is prismatic. After the depth of 140 cm allow waterborne debris of calcium carbonate [21].

Regarding the principal macroelements concentrations from Turulung soil genetic type, it can be observed (Table 1) that the highest Na and Ca concentration were found in 20–40 cm horizon (6737.44 mg/kg respective 6699.34 mg/kg). These values decrease with the soil depth profile so in 60–80 cm interval the values were 5397.27 mg/kg (Na) respective 4400.76 mg/kg (Ca). For Mg the situation is different: the highest values were found in 60–80 cm interval (4696.27 mg/kg) and the lowest in 20–40 (3216.84 mg/kg). It is well known that the great mass of vine active roots is situated in 20–80 cm soil interval [22], were the medium values for Na, Ca and Mg are comparable with the surface (0–20 cm). Kment et al. [23] showed that the Ca and Mg concentration in soil could be influenced by the parent rock causing the high concentration for Ca and Mg in the analyzed soil.

**Table 1:** **The concentration of micro-, macroelements and heavy metals in soil from Turulung area (mg/kg)**

**Depth (cm)**	MLA*	**MLA**	**MLA**	**MLA**	**MLA**	**MLA**	**MLA**	**MLA**	**MLA**	**MLA**
	**-**	**-**	**-**	**-**	**20 mg/kg**	**100 mg/kg**	**20 mg/kg**	**1 mg/kg**	**20 mg/kg**	**15 mg/kg**
	**Na**	**Ca**	**Mg**	**Fe**	**Cu**	**Zn**	**Pb**	**Cd**	**Ni**	**Co**
**0-20**	6497.49 ± 367.99 a	5133.91 ± 434.99 b	3628.81 ± 241.27 bc	2341.40 ± 169.80 ab	433.69 ± 40.82 a	78.25 ± 4.49 a	16.27 ± 0.93 a	0.56 ± 0.30 a	19.23 ± 0.98 a	9.93 ± 1.68 ab
**20-40**	6737.44 ± 245.50 a	6699.34 ± 717.90 a	3216.84 ± 282.26 c	2437.93 ± 269.95 a	471.15 ± 22.56 a	54.58 ± 2.80 b	15.45 ± 1.18 a	0.30 ± 0.01 c	17.70 ± 1.93 a	11.31 ± 1.55 a
**40-60**	6293.69 ± 278.38 a	4904.12 ± 220.65 b	3828.06 ± 139.96 b	2060.90 ± 73.68 b	535.58 ± 84.81 a	74.31 ± 4.40 a	13.72 ± 0.75 b	0.42 ± 0.04 b	12.80 ± 2.55 b	10.28 ± 1.73 ab
**60-80**	5397.27 ± 606.76 b	4400.76 ± 262.31 b	4696.27 ± 301.73 a	2016.83 ± 79.00 b	478.13 ± 67.68 a	70.63 ± 4.37 a	13.63 ± 0.09 b	0.51 ± 0.05 a	15.40 ± 2.59 ab	7.49 ± 0.90 c
**Average**										
	6231.47 ± 374.66	5824.53 ± 408.96	3842.50 ± 240.59	2214.27 ± 148.77	479.64 ± 53.97	69.44 ± 4.02	14.77 ± 0.74	0.45 ± 0.10	16.28 ± 2.01	9.75 ± 1.47
$$ \overline{x}\left(20-80\right) $$										
	6142.80 ± 376.88	5334.74 ± 400.29	3913.72 ± 240.32	2172.89 ± 140.87	494.95 ± 58.35	66.51 ± 3.86	14.27 ± 0.27	0.41 ± 0.03	15.30 ± 2.36	9.69 ± 1.39
**Minimum values**										
	5397.27 ± 606.76	4400.76 ± 262.31	3216.84 ± 282.26	2016.83 ± 79.00	535.58 ± 84.81	54.58 ± 2.80	13.63 ± 0.09	0.30 ± 0.01	12.80 ± 2.55	7.49 ± 0.90
**Maximum values**										
	6737.44 ± 245.50	6699.34 ± 717.90	4696.27 ± 301.73	2437.93 ± 269.95	433.69 ± 40.82	78.25 ± 4.49	16.27 ± 0.93	0.56 ± 0.30	19.23 ± 0.98	11.31 ± 1.55

Average value ± standard deviation (n = 3). Different letters are significantly different for P ≤ 0.05 between depths. The difference between any two values, followed by at least one common letter, is insignificant.

MLA* = maximum limit allowed.

$\overline{x}\left(20-80\right)$ = In the conditions of Romania, most roots vines are grown in soil layers from about 20 to 80 cm [22].

Regarding the microelements analyzed, Cu concentration has higher values than the maximum limit admitted (20 mg/kg). It is well-known that Cu is one of the most studied element in wine-growing regions. It is effective against a high number of crop pests and it is utilised as a fungicide, a bactericide and also as a herbicide [24]. Different Cu formulations are used against grapevine (*Vitis vinifera* L.) downy mildew and they have a secondary effect on grapevine powdery mildew and on a wide range of other grapevine insect pests and diseases [24,25]. The average value of Cu for the soils analyzed is 479.638 mg/kg, with a minimum of 433.69 mg/kg and a maximum of 535.58 mg/kg.

These high values could be an effect of treatments with different Cu products made during the time [9,26], and also as a pollution effect from the two metallurgical factories in Baia-Mare, Romplumb and Cuprom (57 km from Turulung area) which are the main sources of pollution for the city [12] and also for the neighboring agricultural areas.

In one study, the world average values for Cu in soil is reported as 30 mg/kg (from 2 to 250 mg/kg) [27] but other studies showed values from 200 to 500 mg/kg [9,28].

However, concentrations of copper in our study were comparable to those found in other vineyards. For example, studies carried out in vineyards without smelters activities like in Spain, indicate a mean of 35.4 mg/kg [29,30]; 179–579 mg/kg in top soil [31], in France 22–398 mg/kg in top soil (0–30 cm) [28] and 227 mg/kg for the deep horizon (35–40 cm) [9], while in Brazil the vineyard soils presents very high concentrations (up to 3216 mg/kg Cu) [32]. Conversely, in industrially polluted region from Bulgaria, Cu registred a maximum of 72.3 mg/kg [33]; 229.15 mg/kg in old mining area from Banat county [18], and 440–5823 ppm in Baia Mare, Romania, very close to metallurgical smelter factories [12].

Zn values were between 54.58-78.25 mg/kg, under the maximum limit admitted (100 mg/kg). Compared with the data from the literature, the values obtained for Zn in Turulung are slighty higher than the world soil average (50 mg/kg) [27], but appropriate with the values reported in the Castelon region, Spain (76.8 mg/kg) [29]. The Zn content from the Huşi vineyard area (Romania) varied between 43.1 and 103 mg/kg, with an average of 73.9 mg/kg [6]. Regarding the depths from the Brestnik area, Bulgaria, the Zn content decrease from the surface (0–10 cm) to the deep horizon (30–40 cm), namely from 243 mg/kg to 187 mg/kg [33]. The same situation is in our study (78.25 mg/kg at 0–20 cm to 54.58 mg/kg at 20–40 cm) and also in the Champagne region, France (from 318 mg/kg in the topsoil at 5–10 cm to 208 mg/kg in the deep horizon at 35–40 cm) [9].

Pb, Cd, Ni, Co levels were under the maximum limit admitted. Except for Co, the other heavy metals like Pb, Cd and Ni recorded the highest values in the surface (0–20 cm). In the Turulung vineyard soil, Pb recorded a total average of 14.77 mg/kg, varying from a minimum value of 13.63 to a maximum value of 16.27 mg/kg. These values are comparable with those obtained in Huși area, Romania, 19.9 mg/kg [6], Douro basin, Portugal, 28.8 mg/kg [34], and Castelon region, Spain, 56.1 mg/kg [29].

In case of Cd, the average content (0.44 mg/kg); slightly higher than background (0.40 mg/kg) for Cd in world soils, presented by [27] and lower compared with the value registred in the Castelon region, Spain (0.358 mg/kg) [29].

The total average of Ni for the Turulung area soil is 19.23 mg/kg lower than the world soil average value for Ni (40 mg/kg) reported by [27]. Total content mean in the Castelon area, Spain was 19.9 mg/kg Ni [29] and mean value of 28 mg/kg was recorded in the Douro basin, Portugal [34]. In the vineyard from SW of Romania, the Ni content ranges between 13.82 and 31.18 mg/kg for a 0–10 cm depth [11].

Finally, the Co content from the study area varies between 7.49 mg/kg and 11.31 mg/kg, with an average of 9.75 mg/kg. According to [27], based on the data from the literature, the values vary within a range, from 2 to 40 mg/kg Co for world soils, a total content of 7.9 mg/kg for Spain [29], and a average of 12 mg/kg for the Douro basin, Portugal [34].

### Micro-, macroelements and heavy metals in canes and leaves

The concentration levels of micro-, macroelements and heavy metals in aerial parts (canes and leaves) of vinegrape cultivars were determinated. Thus, minerals studied represented the following order: Ca > Na > Mg > Fe > Cu > Zn > Ni > Pb > Co > Cd in canes and Ca > Mg > Na > Fe > Cu > Zn > Ni > Pb > Co > Cd in leaves (Tables 2 and 3). Between macroelements analyzed, Ca with 2521.57 mg/kg in canes, and 3005.27 mg/kg in leaves recorded the highest concentration. These values were lower than in canes from the Turkey (from 5950 ± 50 mg/kg to 10210 ± 120 mg/kg) but in case of Fe concentration, our results (average 400.15 ± 6.72 mg/kg) were higher (from 2.6 mg/kg to 6.8 mg/kg) [35]. Regarding heavy metals levels in canes from study area Turulung (Romania) the averages were: 0.13 ± 0.02 mg/kg- Cd, 2.02 ± 0.19 mg/kg-Pb, 9.87 ± 0.44 mg/kg- Ni, 13.97 ± 1.30 mg/kg- Zn and 55.02 ± 2.54 mg/kg-Cu (Table 2). For Zn the values were lower than reported by [35] in canes from the Experimental Vineyard of Suleyman Demirel University (Isparta, Turkey).

**Table 2:** **The concentration of micro-, macroelements and heavy metals in canes from Turulung area (mg/kg)**

	MLA*	**MLA**	**MLA**	**MLA**	**MLA**	**MLA**	**MLA**	**MLA**	**MLA**	**MLA**
	**-**	**-**	**-**	**-**	**-**	**-**	**-**	**-**	**-**	**-**
**Element Cultivar**	**Na**	**Ca**	**Mg**	**Fe**	**Cu**	**Zn**	**Pb**	**Cd**	**Ni**	**Co**
Fetească albă	1147.55 ± 16.74 b	2593.88 ± 157.91 a	817.40 ± 18.10 c	382.27 ± 4.64 c	39.34 ± 1.80 b	15.04 ± 1.47 a	2.28 ± 0.17 a	0.16 ± 0.02 a	9.81 ± 0.45 b	1.18 ± 0.23 b
Fetească regală	1259.19 ± 22.78 a	2619.36 ± 163.45 a	888.47 ± 10.41 b	400.37 ± 4.89 b	62.05 ± 3.14 a	12.09 ± 1.59 b	1.32 ± 0.19 b	0.13 ± 0.03 a	11.11 ± 0.66 a	1.67 ± 0.20 b
Riesling italian	1027.06 ± 17.81 c	2350.48 ± 123.59 a	958.44 ± 23.68 a	417.82 ± 10.63 a	63.67 ± 2.67 a	14.77 ± 0.83 ab	2.46 ± 0.22 a	0.10 ± 0.02 a	8.69 ± 0.23 c	2.53 ± 0.43 a
**Average**										
	1144.60 ± 19.11	2521.57 ± 148.31	888.10 ± 17.39	400.15 ± 6.72	55.02 ± 2.54	13.97 ± 1.30	2.02 ± 0.19	0.13 ± 0.02	9.87 ± 0.44	1.79 ± 0.28
**Minimum values**										
	1027.06 ± 17.81	2350.48 ± 123.59	817.40 ± 18.10	382.27 ± 4.64	39.34 ± 1.80	12.09 ± 1.59	1.32 ± 0.19	0.10 ± 0.02	8.69 ± 0.23	1.18 ± 0.23
**Maximum values**										
	1259.19 ± 22.78	2619.36 ± 163.45	958.44 ± 23.68	417.82 ± 10.63	63.67 ± 2.67	15.04 ± 1.47	2.46 ± 0.22	0.16 ± 0.02	11.11 ± 0.66	2.53 ± 0.43

Average value ± standard deviation (n = 3). Different letters are significantly different for P ≤ 0.05 between varieties. The difference between any two values, followed by at least one common letter, is insignificant.

MLA* = maximum limit allowed.

**Table 3:** **The concentration of micro-, macroelements and heavy metals in leaves from Turulung area (mg/kg)**

	MLA*	**MLA**	**MLA**	**MLA**	**MLA**	**MLA**	**MLA**	**MLA**	**MLA**	**MLA**
	**-**	**-**	**-**	**-**	**-**	**-**	**-**	**-**	**-**	**-**
**Element Cultivar**	**Na**	**Ca**	**Mg**	**Fe**	**Cu**	**Zn**	**Pb**	**Cd**	**Ni**	**Co**
Fetească albă	235.71 ± 13.51 a	2820.38 ± 100.42 b	1453.90 ± 10.31 a	122.37 ± 2.00 b	47.23 ± 2.10 b	23.94 ± 1.55 ab	4.93 ± 0.04 a	0.58 ± 0.04 c	10.12 ± 0.59 a	0.24 ± 0.02 b
Fetească regală	247.50 ± 11.90 a	3244.99 ± 105.10 a	1381.45 ± 15.22 b	147.54 ± 4.12 a	58.19 ± 4.47 a	27.05 ± 1.39 a	3.12 ± 0.03 b	0.79 ± 0.02 b	9.23 ± 0.90 a	0.33 ± 0.07 b
Riesling italian	188.55 ± 6.57 b	2950.45 ± 74.95 b	1096.26 ± 7.24 c	143.29 ± 6.29 a	42.05 ± 3.63 b	24.62 ± 1.17 b	4.69 ± 0.02 a	0.93 ± 0.02 a	9.06 ± 0.75 a	0.85 ± 0.11 a
**Average**										
	223.92 ± 10.66	3005.27 ± 93.49	1310.54 ± 10.92	137.73 ± 4.14	49.16 ± 3.40	25.20 ± 1.37	4.25 ± 0.03	0.77 ± 0.03	9.47 ± 0.75	0.47 ± 0.07
**Minimum values**										
	188.55 ± 6.57	2820.38 ± 100.42	1096.26 ± 7.24	122.37 ± 2.00	42.05 ± 3.63	23.94 ± 1.55	3.12 ± 0.03	0.58 ± 0.04	9.06 ± 0.75	0.24 ± 0.02
**Maximum values**										
	247.50 ± 11.90	3244.99 ± 105.10	1453.90 ± 10.31	147.54 ± 4.12	58.19 ± 4.47	27.05 ± 1.39	4.93 ± 0.04	0.93 ± 0.02	10.12 ± 0.59	0.85 ± 0.11

Average value ± standard deviation (n = 3). Different letters are significantly different for P ≤ 0.05 between varieties. The difference between any two values, followed by at least one common letter, is insignificant.

MLA* = maximum limit allowed.

**Table 4:** **Concentration of heavy metals in wines from different European viticulture countries (mg/L)**

**Origin**	**Analytical technique**	**Cu**	**Zn**	**Pb**	**Ni**	**Cd**	**References**
Spain	ICP-AES	0.30	0.53	nd	nd	nd	Alvarez et al. [51]
Slovenia	FAAS-ETAAS	0.12	0.50	0.03	nd	0.0003	Kristl et al. [40]
***Romania***	*FAAS*	*0.23*	*0.46*	*0.09*	*0.04*	*ULD*	*This study*
Romania, Moldova	ICP-MS	0.602	0.473	0.043	0.058	nd	Geana et al. [52]
Ukraine	ICP-AES	0.48	0.45	0.03	0.06	nd	Vystavna et al. [3]
Greece	FAAS	0.2-0.6	0.3-3.1	0.018-0.42	nd −2.3	0.006	Galani-Nikolakaki et al. [53]
**OIV**	AAS	1.0	5	0.15	–	0.01	OIV [41]

adapted from Vystavna et al. [3].

nd - was not determined in the referenced study.

ULD - under the limit of detection.

Between heavy metals in leaves (Table 3) the concentration levels of Cu (average 49.16 ± 3.40 mg/kg) and Pb (average 4.25 ± 0.03 mg/kg) were higher than in leaves from the Ucraina (Cu is 9.91 ± 0.9 mg/kg and Pb is 0.99 ± 0.16 mg/kg ) [3], France (Cu is 6.7 mg/kg and Pb is 0.8 mg/kg) [9] and other leafy vegetables grown in contaminated mining areas from Romania (from 0.29 mg/kg to 4.79 mg/kg for Cu and 0.03 mg/kg to 1.79 mg/kg for Pb) [18].

In south-west Romania (Caras Severin County) in which pollution was generated for many decades by extractive and metallurgical industry [11] recorded for Pb a range of 0.18 - 6.56 mg/kg, for Ni 0.37-2.58 mg/kg, and for Cd 0.10-1.10 mg/kg. These values are similar whith our results.

Zn was found in the average of 25.20 ± 1.37 mg/kg (Table 2) less than those from France (average Zn content is 29.3 mg/kg) [9] and Ucraina (average Zn content is 28 mg/kg) [3].

### Heavy metals in grape juice (must) and wine

**Figure 1:**
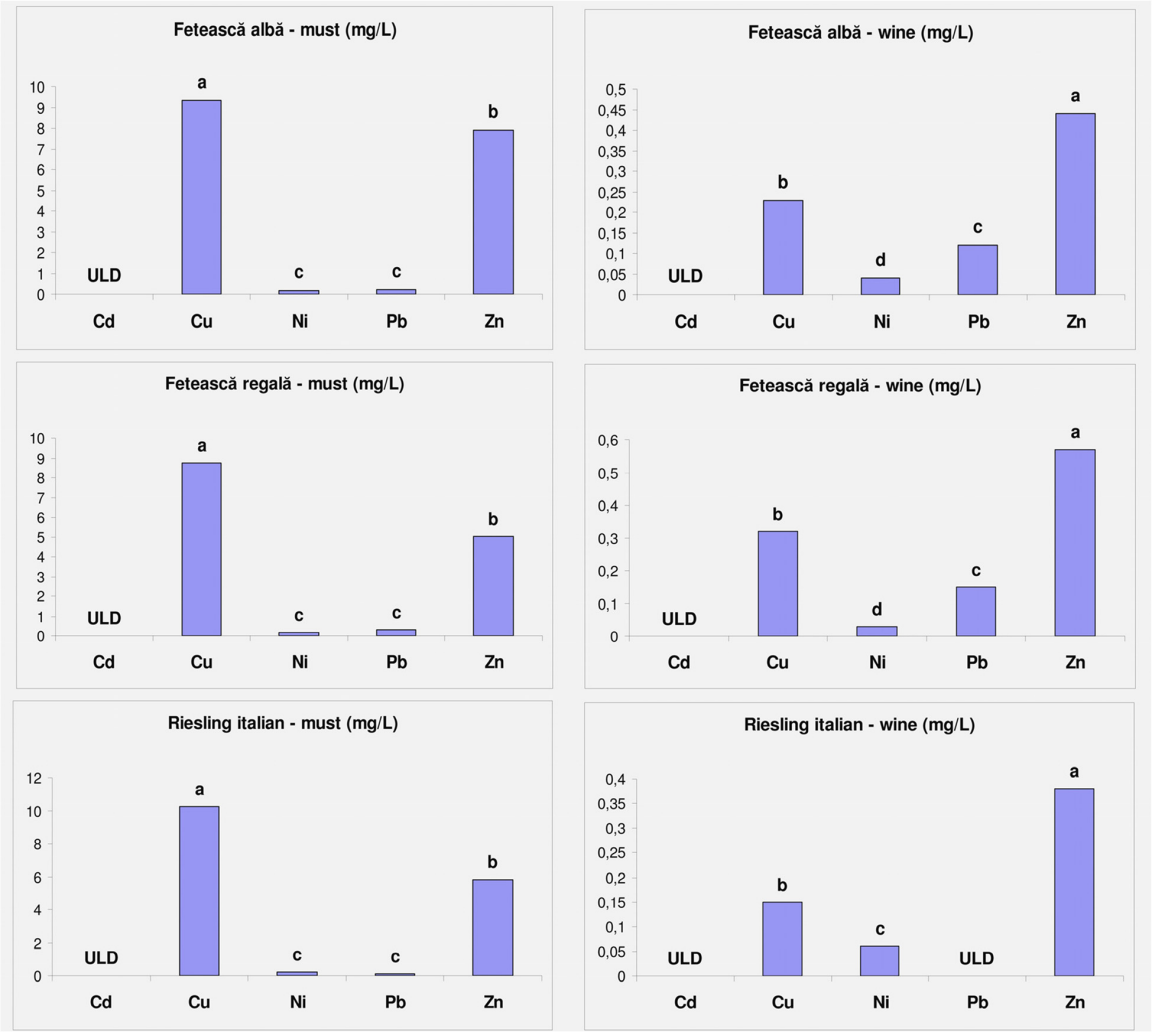
Concentration of heavy metals in must and wine (mg/L); ULD = under the limit of detection.

The concentrations of heavy metals in grape must samples decrease in the order Cu > Zn > Pb > Ni > Cd, for majority of winegrape cultivars studied. The concentration levels of Zn in white must ranged from 5.02 mg/L in Fetească regală to 7.93 mg /L in Fetească albă, and Cu was from 8.77 mg/L mg in Fetească regală to 10.22 mg/L in Riesling italian (Figure 1).

The higher concentration levels of Cu were observed in Riesling italian compared to Fetească albă and Fetească regală, Zn level are higher in Fetească albă, while Ni and Pb present higher concentrations in Fetească regală, which can be linked to different accumulation patterns in the cultivars. A previous study by Ko et al. [36] reported the variation of accumulation patterns of trace metals depending on the type of cultivar.

The results represent a higher heavy metals content to the grape juice from Ukraine [3], Brasil [37], Portugal [38] and other vinegrowing area from Romania [39]. It was expected to obtain higher concentrations for heavy metals analyzed because the study area (Turulung) is located close to Baia Mare.

The concentration level of Cu decreased from 9.32 mg/L in grape juice to 0.23 mg/L in the wine from Fetească albă cultivar, from 8.77 mg/L in grape juice to 0.32 mg/L in the wine from Fetească regală cultivar and from 10.22 mg/ L in grape juice to 0.15 mg/L in the wine from Riesling italian cultivar. The same decreasing trend is observed also for the other elements analyzed (Ni, Pb and Zn) at all three cultivars tested. Particularly, cv. Fetească regală recorded higher concentration levels for all elements (except Ni) and cv. Riesling italian the lower (except Ni).

Cu, Pb, Ni and Zn concentration levels decreased in wine compared to grape must, possibly forming insoluble components that can be removed through sedimentation together with yeasts and lees during fermentation [40]. Cd was for all cultivars under the limit of detection (in must and wines).

By comparing the values for heavy metals in wines found in literature (Table 4) with the concentration levels of Cd, Cu, Ni, Pb, and Zn in young white wines obtained in Turulung area (NW Romania), we can see that all three wines were similar with the values found in some European countries. Cd, Cu, Zn and Pb in Romanian wine were below the recommended health limits of the International organisation of Vine and Wine (OIV) [41].

### Traceability of heavy metals in system soil-grapevine-wine

The mobility ratio (MR) in *Vitis vinifera* L. was used by Serbula et al. [42] and Vystavna et al. [3] to determine the ratio of heavy metals concentration levels (C_plant_, mg/kg) in plant parts (leaves and grapes) to the concentration level of the acid-soluble metal fraction (C_soil-m_, mg/kg) in the top-soil MR = C_plant_ / C_soil-m_.

In other studies accumulation ratio (AR) was used to determine the ratio of metals concentration in plant parts (leaves and grapes) to its pseudo-total concentration (C_soil-t_, mg/kg) in the top-soil (AR = C_plant_/ C_soil-t_) [3,9].

In our study transfer factors (TF) were calculated to reveal traceability and bioavailability of heavy metals (Cu, Zn, Pb, Cd, Ni), in system soil-grapevine-wine. Thus were calculated: TF_cs_ = C_canes_/C_soil_, TF_lc_ = C_leaves_/C_canes_, TF_mc_ = C_must_/C_canes_, TF_wm_ = C_wine_/C_must_ as the ratio between heavy metal concentration of: canes-soil; leaves-canes; must-canes respectively wine-must. The results obtained are shown in Table 5.

**Table 5:** **Transfer factors in system soil-grapevine-wine (mg/kg and mg/L); STDV= standard deviation; RSD%= relative standard deviation**

**TF**_**cs**_ ** = C**_**canes**_ **/C**_**soil**_ ** c**	**Canes**					
**Cultivar**	**Cu**	**Zn**	**Pb**	**Cd**	**Ni**	
Fetească albă	0.079	0.226	0. 1 60	0.400	0.641	
Fetească regală	0.125	0.182	0.093	0.325	0.726	
Riesling italian	0.129	0.237	0.173	0.250	0.568	
**AVERAGE**	**0.111**	**0.215**	**0.142**	**0.325**	**0.645**	Ni > Cd > Zn > Pb > Cu
**STDEV**	**0.027**	**0.029**	**0.043**	**0.075**	**0.079**	
**RSD%**	**24.724**	**13.625**	**30.340**	**23.077**	**12.271**	Pb > Cu > Cd > Zn > Ni
TF_Ic_ = C_leaves_/C_canes_	Leaves					
Cultivar	Cu	Zn	Pb	Cd	Ni	
Fetească albă	1.201	1.592	2.162	3.625	1.032	
Fetească regală	0.938	2.237	2.364	6.077	0.831	
Riesling italian	0.660	1.561	1.907	9.300	1.043	
**AVERAGE**	**0.933**	**1.797**	**2.144**	**6.334**	**0.968**	Cd > Pb > Zn > Ni > Cu
**STDEV**	**0.270**	**0.382**	**0.229**	**2.846**	**0.119**	
**RSD%**	**28.951**	**21.254**	**10.685**	**44.936**	**12.314**	Cd > Cu > Zn > Ni > Pb
TF_mc_ = C_must/_C_canes_	Must					
Cultivar	Cu	Zn	Pb	Cd	Ni	
Fetească albă	0.237	0.527	0.101	0.000	0.017	
Fetească regală	0.141	0.415	0.242	0.000	0.018	
Riesling italian	0.161	0.370	0.041	0.000	0.022	
**AVERAGE**	**0.180**	**0.438**	**0.128**	**0.000**	**0.019**	Zn > Cu > Pb > Ni
**STDEV**	**0.051**	**0.081**	**0.104**	**0.000**	**0.002**	
**RSD%**	**28.153**	**18.471**	**80.934**		**12.837**	Pb > Cu > Zn > Ni
TF_wm_ = C_wine/_C_must_	Wine					
Cultivar	Cu	Zn	Pb	Cd	Ni	
Fetească albă	0.025	0.055	0.522	0.000	0.235	
Fetească regală	0.036	0.114	0.469	0.000	0.100	
Riesling italian	0.01 5	0.065	0.000	0.000	0.316	
**AVERAGE**	**0.025**	**0.078**	**0.330**	**0.000**	**0.217**	Pb > Ni > Zn > Cu
**STDEV**	**0.01 1**	**0.031**	**0.287**	**0.000**	**0.109**	
**RSD%**	**43.186**	**39.888**	**86.974**		**50.246**	Pb > Ni > Cu > Zn

In the case of transfer factor leaves-canes (TF_lc_) the highest values were obtained for Cd and Pb. Conversely the analysis of average contents in heavy metals from canes, leaves, must and wines showed that the highest average contents for Pb (2.144 mg/kg) and Cd (6.334 mg/kg) were found in leaves.

This highlights the behavior of vine regarding to the aggression of toxic metals like Pb and Cd. The results show that generally, in response to the accumulation of toxic metals, vine will direct these metals to the leaves in order for their elimination at the end of the growing season.

The heavy metals medium content analysis in must and wine showed lower values compared with leaves and canes. Conversely the wine metal contents (from 0.000 to 0.333 mg/L) are lower than in must (from 0.000 to 0.438 mg/L).

From Table 5 it can be observed that the transfer coefficients must-canes (TF_mc_) and wine-must (TF_wm_) has subunits values, this fact can be explained through the intent of vine to accumulate metals, especially toxic metals at aerial parts level (leaves, canes). This fact showed that vine has specific mechanisms to block toxic metals accumulation (like Pb and Cd) in aerial parts and in their transfer to the berries (grape juice). This finding is in agreement with other studies [9,23,43].

The physico-chemical and biological processes which take place in must transformation to the wine, generate the reduction of heavy metals. This fact it is showed in metal lower values in wines compared with the values found in must or by the subunits values of the transfer factors.

## Conclusions

The results of our study showed diverse patterns of Na, Ca, Mg, Fe, Cu, Zn, Pb, Cd, Ni and Co accumulation in the soil (0–80 cm), canes, leaves, grape juice and wines of three winegrape cultivars from the Turulung area, NW Romania. In soil, for all the minerals studied the results were under the maximum limit admitted [44], except for Cu (average 479.64 mg/kg while MLA = 20 mg/kg). These raised values obtained could be an effect of different copper treatments aplied in vineyard against downy mildew and as an industrial pollution effect. In canes and leaves Cu, Zn, Pb, Cd, Ni had higher concentration levels compared with must and wine. Conversely the wine metal accumulation are lower than in must. The transfer factors calculated showed *Vitis vinifera* L. specific avoidance strategy for preventing toxicity. Regarding the high amount of Cu in the soil vineyard, it was demonstrated that the resulting wine did not present Cu levels above maximum limit allowed (limit recommended by OIV).

## Experimental

### Study area

**Figure 2:**
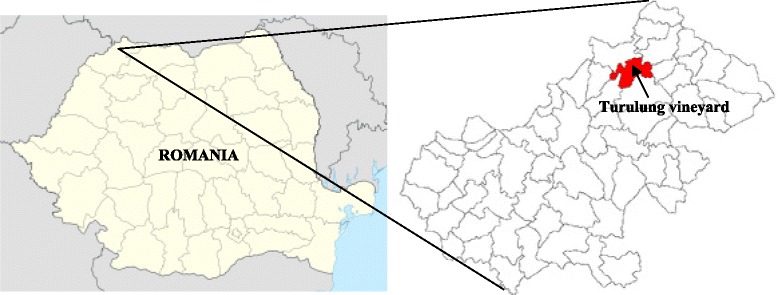
Location of the study area (Turulung vineyard).

The study area Turulung is located at 47° 56′ North, 23° 5′ East, in the Satu Mare county, Romania at 57 km northwest from Baia Mare city. The European road E 81 passes through Turulung, connecting it to the Republic of Ucraina through the Halmeu Customs (Figure 2). As geological-morphological unit Satu Mare county is situated at the eastern edge of the Pannonian Basin, and is separated from Transylvanian Basin by the volcanic mountains Gutâi-Igniș and Codrului Peak mountains [45]. Turulung vineyard has an altitude of 133 m, annual precipitations 687 mm, and annual average temperature 10.3°C [46].

The study area (4 ha) is used for the growing of two domestic vine cultivars Fetească albă and Fetească regală, and one international vine cultivar Riesling Italian for the obtain of wines with geographical indication “Sătmar Hills”, Romania. All vines were planted since 2007, and the vine plantation was organized with 2.2 x 0.9 m distance between rows and plants. Vines were pruned according to the Guyot system and were grown on speliers.

Type of soil is Preluvosoil (EL). This is Luvisols characterized morphologically by: A ocric horizon presence or mollic horizon (Ao, Am) followed by argic intermediate horizon (Bt) with color values exceeding 3.5 at wet material from the top and base saturation level (V%) over 53%. Preluvosoil typical profile has a slightly shorter than the other soils found in the complex because it meets at the southern slopes, better warm or on a microrelief with good drainage and parent material rich in alkaline elements [21].

### Sampling and samples preparation

The sampling was carried out from August - October 2013 as follows: the soil samplings were done at Turulung in August 2013 on four depths (0–20 cm, 20–40 cm, 40–60 cm, 60–80 cm); sampling of leaves were done in August 2013; sampling of grapes were made in September 2013 and sampling of canes were conducted in October 2013. The study included sampling such as: soil of the vineyard; canes and leaves of Fetească albă, Fetească regală and Riesling Italian cultivars; the first cold pressed grape juice used for the winemaking and young wine after the alcoholic fermentation. All samples were taken in triplicates from the defined experimental plot.

**Soil sampling** was conducted in accordance with the recommendations of the Order of the Ministry of Agriculture, Food and Forests no. 223, updated and published in Romanian Official Monitor No. 598/13 August 2002 [47]. Soil samples (3 samples/depth) were collected from a depth of 0–20 cm, 20–40 cm, 40–60 cm, and 60–80 cm using a handle steel soil sampler. Agrochemical sampling depth of 0–20 cm is performed after a prior removal of dust, roots, leaves or other residues from the surface. The amount of collected sample is between 0.5 to 1 kg. Each sample is placed in properly labeled plastic bags, closed tightly, ISO 11464/1994. The essential point in the determination of the depth of soil sampling is that the samples should be representative for the depth explored by the roots and also by the fertilizers and amendments incorporation level [48]. Preparation of the samples analysis consisted in removal of foreign matters, milling and sieving of the soil. The soil drying was carried out at a temperature of 105°C using an oven model FD 53 Binder. Subsequently samples pulverization and homogenization was performed using an automatic mortar Resh 110 Germany. 50 g of homogenised samples were prelevated for further analysis [49]. For disaggregating soil samples the working protocol ISO 11466/1999 was used. An amount of 0.2 - 0.5 g dried and milled material was put into 12 mL aqua regia (9 mL HCl +3 ml HNO_3_) and after 15 minutes the mineralization was performed using a microwave Berghof MWS-2, set in 2 steps (at 180°C and 100°C). After disaggregation, the samples were filtered through a 0.45 mm filter and brought to a final volume of 100 mL. For dilution of soil samples was used 50 mL ultrapure water. Samples filtration and dilution was done according with ISO 11466/1999.

**Sampling of leaves and canes** was done randomized, from 10–12 representative vines for each cultivar on the same plot. From each choosing vine were picked up 2–4 cane pieces (25 cm) and 2–4 leaves (from different parts of canopy), that make up the sample composed. Samples of plant material which suffered injuries caused by insects or mechanical damage have been removed. After this, the plant material samples were placed in sealed plastic bags, and immediately transported to the laboratory for analysis. All vegetable samples were washed (2–3 times) with double distilled water to remove soil pollutants. After washing, vegetable samples were oven dried at 80°C to constant weight. The dried samples were ground, passed through a 2 mm sieve, mineralized 8 h at 550°C and stored at ambiental temperature before analysis [18]. The micro-, macroelements and heavy metals contents in vegetable samples were carried out in HNO_3_ solution resulted by plants ash digestion [18,20]. Each sample solution was made up with dilute HNO_3_ (2 mol/L) to a final volume of 100 mL and analyzed by flame atomic absorbtion spectrometry (FAAS). Filtration and dilution of samples was done respecting ISO 11466/1999 (EU rules).

Grape samples (5 kg/cultivar) were collected for each cultivar from 10–12 vines. The grapes were placed in the lower third, middle and top of each vine and grapes were exposed to sun and shade [50]. In this way can achieve better homogenization of the sample grapes. Fetească regală (3 samples), Fetească albă (3 samples) and Riesling Italian (3 samples) **grape juices (must)** were cold pressed manually. Before the analysis, each juice sample (50 mL) was diluted in different proportions using distilled water. **Young wines** (3 samples/cultivar) were analysed from the corresponding vineyard in November 2013, and measured without the pretreatment and digestion. The wines were produced in the laboratory conditions (micro-vinification) in the same year (2013) as the grapes sampled.

### Chemical analysis

The soil samples, leaves, canes, musts and wines were analysed by FAAS (Perkin Elmer AAnalyst 800, Shelton, USA). Flame-AAS is the official method of analysis for the determination of trace elements with relatively high concentrations according to EU regulations. The analysis precision was usually very good, being on average above 1% for all the elements considered at the mg/L or mg/kg concentration level [5].

All reagents used were of analytical grade (Merck, Germany). Stock standard solutions were prepared weekly or whenever an error is suspected due to these solutions. There were used only standard solutions with commercially distilled water (Merck) at a concentration of 1000 mg/L for mineral elements which will be determined. High purity water from Barnastead Easypure RoDi model D13321, England apparatus was used to prepare the standard solutions. The intermediate solutions were stored in polyethylene bottles and glassware was cleaned by soaking in 10% v/v HNO_3_ for 24 hours and rinsing at least three times with ultrapure water. For quality control purpose, blanks and triplicates samples (n = 3) were analyzed during the procedure. The variation coefficients were under 10% and detection limits (mg/L) were determined by the calibration curve method. LOD (Limit of detection) and LOQ (Limit of quantification) limits were calculated according to the next mathematical formulas: LOD = 3 SD/s and LOQ = 10 SD/s (SD = the estimation of the standard deviation of the regression line, and s = slope of the calibration curve). The results obtained are shown in Table 6.

**Table 6:** **Instrumental conditions for the determination of each element (FAAS technique)**

**Element**	**Wavelength**	**Slit**	**Correlation**	**Flame**	**Background**	LOD*	LOQ**
	**(nm)**	**(nm)**	**coefficient**	**(2300°C)**	**correction**	**(mg/L)**	**(mg/L)**
Ca	422.7	0.7	1.000000	Air-acetylene	-	0.092	0.306
Mg	285.2	0.7	1.000000	Air-acetylene	Deuterium	0.190	0.633
Fe	248.3	0.2	0.999972	Air-acetylene	Deuterium	0.110	0.366
Zn	213.9	0.7	0.999999	Air-acetylene	Deuterium	0.018	0.059
Cu	324.8	0.7	0.999979	Air-acetylene	Deuterium	0.017	0.056
Ni	232.0	0.2	0.999920	Air-acetylene	Deuterium	0.020	0.066
Co	240.7	0.2	0.999900	Air-acetylene	Halogen	0.120	0.399
Pb	283.3	0.2	0.999853	Air-acetylene	Deuterium	0.051	0.166
Cd	228.8	0.7	1.000000	Air-acetylene	Deuterium	0.028	0.093
Na	589.0	0.2	1.000000	Air-acetylene	-	0.012	0.039

*Detection limit.

**Quantification limit.

- Not used background correction.

### Statistical analysis

The data were expressed as mean ± standard deviation (SD) of three replications for each sample analyzed. In order to determine the significance differences among values, analysis of variance (ANOVA) and Duncan multiple range tests (MRT) were performed (PoliFact 2010 ANOVA and Duncan’s Test PC Program). Significance of difference was defined at the 5% level (p < 0.05).

## Abbreviations

FAAS: Flame atomic absorption spectrophotometry
O.I.V: International organization of wine and vine
EL: Preluvosoil
LUV: Luvisols
MR: Mobility ratio
C: Concentration
TF: Transfer factor
MLA: Maximum limit allowed
NW: North-West
ISO: International Organization for Standardization
EU: European Union
MV: Millivolts
